# The Effect of Eye-Feedback Training on Orienting Attention in Young Adults With Sluggish Cognitive Tempo

**DOI:** 10.3389/fpsyt.2020.00184

**Published:** 2020-03-17

**Authors:** Kiho Kim, Youna Lee, Jang-Han Lee

**Affiliations:** ^1^Department of Psychology of Counseling, Sejong Cyber University, Seoul, South Korea; ^2^Department of Image Engineering, Chung-Ang University, Seoul, South Korea; ^3^Clinical Neuro-Psychology Lab., Department of Psychology, Chung-Ang University, Seoul, South Korea

**Keywords:** sluggish cognitive tempo (SCT), attention training, eye-feedback, eye-movements, attention network test (ANT)

## Abstract

Sluggish cognitive tempo (SCT) is a kind of attentional symptoms characterized by symptoms of slowness in behavior or in thinking. The aim of the present study was to develop a preliminary attention training program based on real-time eye-gaze feedback using an eye-tracker. A total of 38 participants with SCT were randomly assigned to one of following two conditions: eye-feedback (*N* = 19; Mean Age = 21.21; range 18–26) or control (*N* = 19; Mean Age = 20.68; range 18–25). The participants in the eye-feedback condition received three repeated trainings on the modified version of the Posner's spatial cueing test; we also used real-time constant eye-gaze feedback designed to lead the participants to quickly and accurately engage and to disengage, with pre- and post- measurement of eye-movements (overt attention) and the revised attention network test (ANT-R; covert attention). The participants in the control condition received three repeated same trainings without any feedback, with pre- and post-measurement of eye-movements measure and ANT-R. The results revealed that the eye-feedback group showed a greater improvement in engaging and disengaging attention through the overt attention measure than the control group. The eye-feedback group also showed a greater increase only in the orienting network related to disengaging attention in the covert attention measure compared to the control group. These results suggested that the eye-feedback can be meaningfully used in attention training to enhance the efficiency of attention in clinical settings.

## Introduction

SCT is a kind of attentional construct characterized by symptoms of slowness in behavior or in thinking, difficulty initiating and sustaining effort, hypoactivity, daydreaming, forgetfulness, and confusion in thinking (1, 2). Despite the growing body of research on the treatment for SCT symptoms using pharmacological and behavioral treatments (3, 4), there remains a need for research of intervention targeting attentional difficulties among individuals with SCT. Although the underlying mechanism in SCT remains unknown, it has been suggested that SCT is not primarily a disorder of executive functioning such as attention-deficit/hyperactivity disorder (ADHD); rather, it is associated with poor efficiency in orienting network (5, 6). Given that since the networks of attention are considered as skills that can be improved through practice (7, 8), attention training is not only a clinical intervention, but also an educational program. Previous research has demonstrated a brief training (e.g., 77 min) (9) has a significant effect on attention, and it appears that this effect can transfer to non-trained cognitive skills, such as academic performance (10, 11). In addition, attention training was found to be more effective for individuals with attentional problem such as ADHD, brain injury, and schizophrenia, when it was adaptive condition, and when it targeted the orienting networks (7, 12).

Deriving from the known ability of humans to change their behavior in order to adapt to the environment, known as behavioral plasticity (13, 14), the rationale for attention training is based on the assumption that efficiency of attention could be enhanced after repetitive practice (8, 15). The underlying mechanism of behavioral plasticity is that, if behavior changes, there should also be changes in organization or properties of the neuroanatomical networks in charge of producing the behavior (16). Similarly to any motor skill, oculomotor performance could be improved through practice of saccades, short and rapid eye-movements used to move the fovea to an object or place of interest for detailed visual exploration (17). The saccadic eye-movements (SEM) could be considered a cognitive parameter to evaluate visual attention, and can be divided according to two types of saccades. First, the pro-SEM is a redirection of the gaze to a visual stimulus in the environment related to the alerting and orienting networks; second, the anti-SEM is a voluntary gaze redirection in the opposite direction of a visual stimulus to inhibit automatic saccades related to executive control network (18). Several studies demonstrated that repetitive training of the SEM produces not only behavioral variations, such as decreasing in the latency of saccades and increasing in the saccadic accuracy, but also changes in the neural activity of the ocular motor network, such as supplementary eye field, frontal eye field, superior parietal lobe, cuneus, and superior colliculus (13, 19, 20). Furthermore, a significant post-training improvement in anti-SEM related to executive control was observed in individuals with ADHD who had difficulties in impulsivity control and goal achievement (21). Therefore, it is necessary to explore whether or not attention training using Pro-SEM related to orienting network can be beneficial for individuals with SCT with poor efficiency in that network.

According to the attentional network theory, there are three basic components of attention: alerting, orienting, and executive control (22, 23). The alerting network refers to the ability to prepare and maintain response readiness. The alerting functioning is critical for optimal performance in tasks involving higher cognitive functions. The executive control network accounts for the ability to control goal-directed behavior, detect target and errors, resolve conflicts, and inhibit automatic responses. The orienting network takes charge of the ability to selectively engage in specific information among various inputs and to disengage from what is currently focused on in order to attend another stimulus. Dysfunction of orienting network is related to not only attentional measures, but also SCT symptoms occurring in daily life such as slowness in behavior or in thinking, difficulty in initiating and sustaining effort, daydreaming, and confusion in thinking (5, 6). This makes it imperative to understand the underlying mechanism of orienting network and to repetitively use a task based on the theoretical evidence in order to enhance orientation network in individuals with SCT. One such method of estimating the orienting of attention is the Posner spatial cueing paradigm (24). This paradigm uses a covert attention task based on the RT (i.e., moving to a spatial location without eye-movements). However, the controversy remains as to whether covert shifts of attention are possible without eye-movements (25) and whether or not training covert attention could yield benefits for overt attention and vice versa (26). Also, there is an increasing necessity to directly assess each element of the orienting network. Therefore, in order to directly measure engagements, disengagements, and shifts of orienting network, in the present study, we used the modified the Posner spatial cueing paradigm and an overt attention task (i.e., moving to a spatial location with eye-movements) based on an eye-tracking system. Our primary goal was to establish whether repeated training of the modified task could enhance deficit of orienting network on covert and overt attention in individuals with SCT.

This study tested whether the effects of real-time constant eye-gaze feedback during repeated training of the modified the Posner spatial cueing task could improve orienting network in individuals with SCT. An adaptive attention training that provides performance feedback throughout the training is known to be more effective than a non-adaptive attention training where repetition of the same procedure without the feedback is conducted throughout training (7, 27). Previous research demonstrated that feedback could improve cognitive or behavior performances by reducing uncertainty and providing information to focus on correct response, incorrect response, or both (28, 29). In particular, given that individuals generally have poor metacognitive information of their own eye-movements, providing feedback on eye-movements could be helpful. Therefore, it may be effective to provide constant real-time feedback on eye-movements according to individuals' response in attention training. The results of previous studies highlighted that real-time constant feedback on eye-movements could modify the oculomotor behavior and reinforce intrinsic oculomotor perception (30). In addition, in the studies that used constant eye-gaze contingent feedback, training was found to lead to an efficient implementation of attentional control (31).

The aim of the present study was to investigate whether repeated attention training targeting orienting network could enhance dysfunction of a certain network in individuals with SCT. To this end, a modified Posner spatial cueing paradigm that provides real-time constant eye-gaze feedback was used. The task was designed for the participants to quickly and accurately respond to orienting network; thereafter, we compared the training effects after repetition of attention training between individuals with SCT who received real-time constant eye-gaze feedback (eye-feedback condition) and those who received no feedback (control condition). It was hypothesized that the SCT group in eye-feedback condition would show a greater improvement in orienting network on both covert and overt attention than the SCT group in control condition. Additionally, considering that attention training is generally dull and repetitive, which makes it an unpleasant and unengaging experience for the trainee, particularly if s/he has attentional difficulties (32, 33), we modified the task to a game so that to make it a more engaging experience for the participants.

## Materials and Methods

### Participants

The sample size was calculated using G^*^power 3.1 (34). A total of 36 participants was required to demonstrate the medium effect size of Cohen's *f* = 0.25, a power (1 – β) of 95%, and an alpha of 0.05. On the basis of this estimate, a conservative goal of 42 participants was established allowing a drop out of 15%. Prior to the experiment, as an initial screening measure for SCT, a total of 1,098 adults completed the Barkley Adult ADHD Rating Scale IV (BAARS-IV) (35). Candidate participants were recruited through advertisements in psychiatric clinics, online communities of individuals with attentional problems, and an Internet bulletin board of several universities in Seoul, Korea. Based on the previous recommendations concerning the inclusion criteria (1, 35, 36), a threshold corresponding to the 95 percentiles of five or more symptoms was used to identify SCT or ADHD. A total of 90 participants completed the structured clinical interview for DSM-5 (SCID-5) (37) by clinical psychologists to determine diagnosis and their eligibility to participate. Exclusion criteria in the present study were as follows: (1) problems with intellectual ability; (2) history of head injury; (3) history of drug exposure; (4) diagnosis with ADHD; and (5) diagnosis with other neurological or psychiatric disorders. As a result, 22 individuals with SCT & ADHD (the SCT & ADHD group with at least five or more of symptoms of both SCT and ADHD), 23 individuals with ADHD only (the ADHD only group with at least five or more of symptoms of ADHD but not more than five symptoms of SCT) were excluded in the final sample of the present study. Finally, after controlling for ADHD symptoms, a total of 45 young adults who met inclusion criteria of SCT (SCT-only group with at least five or more symptoms of SCT but not more than five symptoms of ADHD) were asked to participate in the experiment. They were randomly assigned by simple randomization procedure to one of following conditions: the eye-feedback (EF) or the control condition. Randomization sequence was created using Microsoft Excel 2007 for windows (Microsoft, Redmond, WA, USA) with a ratio of 1:1 allocation by an independent research assistant who is not involved in the study. Of all participants, seven participants were excluded for the final data set: three because their eye-movements were not measured due to an eye-tracking device malfunction, two because half of the data was missing due to errors of the eye-feedback task, and two due to the drop-out. Finally, a total of 19 participants in the EF condition received three repeated training sessions with real-time constant eye-gaze feedback. Furthermore, a total of 19 participants in the control condition received three repeated attention training sessions without any feedback.

### Questionnaires

#### The Barkley Adult ADHD Rating Scale IV (BAARS-IV)

The BAARS-IV, previously developed to assess the levels of ADHD and SCT (35) and validated (38), contains 18 items that are consistent with DSM-5 criteria for ADHD and 9 items that target the symptoms of SCT. Using a four-point scale (1 = not at all; 2 = sometimes; 3 = often; 4 = very often), the participants responded to each item with reference as to how often each statement best described their behavior in the past 6 months. Korean version of the BAARS-IV was used to classify young adults with clinically elevated SCT symptoms (39). In the present study, Cronbach's α values were 0.90, 0.80, and 0.90 for the ADHD inattention, ADHD hyperactive-impulse, and SCT, respectively.

#### The Adult Concentration Inventory (ACI)

The ACI, developed for a new adult self-report measure of SCT (40), includes 10 items identified in a recent meta-analysis as optimal for the assessment of SCT symptoms (2). Korean version of the ACI was used to measure the severity of SCT symptoms repeatedly (39). These items were rated on a four-point scale (0 = not at all; 1 = sometimes; 2 = often; 3 = very often) with reference to the past 6 months. In this study, Cronbach's α was 0.85.

#### The Beck Depression Inventory-Second Edition (BDI-II)

The BDI-II, which was developed to assess the levels of depression (41), includes 21 items associated with physical and cognitive symptoms of depression. These items were rated on a four-point scale (0 = not at all; 1 = mildly; 2 = moderately; 3 = severely) with reference to 1 week. Korean version of the BDI-II (42) was used in this study. In this study, Cronbach's α was 0.89.

#### The Beck Anxiety Inventory (BAI)

The BAI, developed to assess the levels of anxiety (43), includes 21 items related to physical and cognitive symptoms of anxiety. These items were rated on a four-point scale (0 = not at all; 1 = mildly; 2 = moderately; 3 = severely) with the reference to 1 week. Korean version of the BAI (44) was used in this study. In the present study, Cronbach's α was 0.89.

### The Revised Attention Network Test (ANT-R)

**Figure 1:**
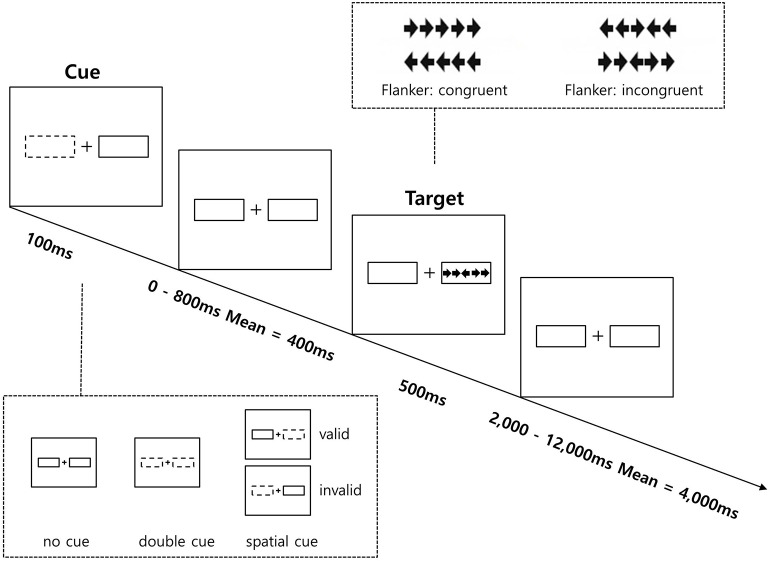
Schematic of the Revised Attention Network Test (ANT-R). Participants make a response to the target's direction.

The ANT-R was administered to measure the efficiency of three attentional networks: alerting, orienting, and executive control (45). The ANT-R is a computerized task consisting of three cue conditions (no-cue, double-cue, and spatial-cue) and two target conditions (congruent, incongruent). Further details on the ANT-R is provided in Figure 1.

Participants were asked to determine as quickly and accurately as possible the direction (left or right) of a central arrow (the target) located in the middle of a horizontal line presented either at the left or right of the screen. The target arrows appeared at one of two locations to the left and right sides of a central fixation cross for 500 ms, and either one or both of the boxes were flashed as a cue by briefly changing its color from black to white for 100 ms prior to the target arrow's appearance. There were three cue conditions: (1) no-cue (no flash before the target appeared; 12 trials); (2) double cue (both cue boxes flashed before the target appeared, so the cue provided temporal, but not spatial information for the target; 12 trials); and (3) spatial cue (one cue box flashed before the target appeared, so the cue provided temporal and spatial information for the target; 48 trials). Additionally, in order to estimate disengagement, shift, and engagement, 75% of the 48 spatial cues were valid (in the same location as the upcoming targets; 36 trials), while 25% were invalid (in the opposite location as the upcoming targets; 12 trials). After a variable duration (0, 400, or 800 ms), the participants were asked to identify the direction of the center target arrow flanked on each side by two flanker arrows pointing either in the same direction as the center target arrow (congruent), or in the opposite direction (incongruent) with same probability (50% each). They responded by pressing the corresponding button (left or right) on the keyboard. The duration between the offset of the target arrows and onset of the next trial varied systemically between 2,000 and 12,000 ms. The response time window was 1,700 ms after the onset of the target and flankers.

Prior to the analysis of the ANT-R, mean RT and error rate for each condition were calculated. Error trials and RTs below 200 ms and above 1,700 ms were excluded from the calculations of mean RT and attentional effects (45). Three networks of attention were considered for the ANT-R in the present data analysis. First, the alerting network represents the benefit of the target response speed by calculating the difference between the no cue and double cue conditions. Second, in the ANT-R, the orienting network could be separately measured as: (1) the engaging index (orienting network in the original ANT) represents the benefit of target response under valid cue condition because of orienting and engaging is measured by the difference between double cue and valid cue conditions; (2) the disengaging index represents the cost of disengaging from invalid cue and is measured by the difference between the invalid cue and double cue condition; (3) the validity index represents the cost of disengaging, and move operation is measured by difference between invalid cue and valid cue conditions. Third, the executive control network represents the flanker conflict effect measured by the difference between incongruent and congruent conditions.

### The Eye-Feedback

**Figure 2:**
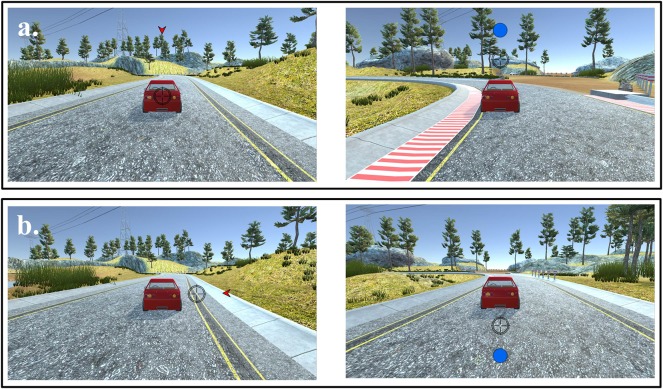
Sample frames from a block of trials with eye-feedback condition. Stimuli (car, arrowhead cue, and circle target) and eye-gaze point used in eye-feedback condition: **(a)** Valid condition: cues were informative about where the target would appear. **(b)** Invalid condition: cues were uninformative about where the target would appear.

**Figure 3:**
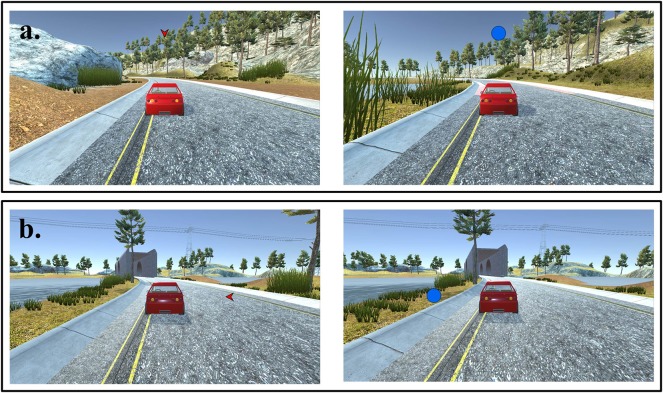
Sample frames from a block of trials with control condition. Same stimuli as eye-feedback condition used in control condition except for eye-gaze point: **(a)** Valid condition: cues were informative about where the target would appear. **(b)** Invalid condition: cues were uninformative about where the target would appear.

The eye-feedback task was developed as a game-based task using Unity 3D game engine to enable users training continuously and repeatedly. The goal of the game was to provide appropriate feedback to the user and improve orienting of attention, based on the fundamental features of the Posner spatial cueing paradigm. The user's control over the game was done only through an eye-tracking device. It allows the user not only to see their own eye-gaze point in real time, but also to receive feedback on their response speed in relation to the movement of attention. The task is a simple form of car racing game: the car in the game acts as a fixation, and targets (blue or orange circles) and cues (red triangles) appear in colors and forms that are visually recognizable to users. Further detail about the task is provided in Figures 2, 3.

The eye-feedback task is a computerized task consisting of two cue conditions (valid-cue, invalid-cue) and four locations (0°, 90°, 180°, 270°). Targets in blue or orange circles were displayed at four possible locations (separated by 90°) for 500 ms, while cues in red inward pointing triangles were displayed for 500 ms prior to the target circle appearing. In order to estimate and improve both engagement and disengagement of attention, the eye-feedback task was made up of two cue conditions with the same probability: (1) 50% of valid cues (in the same location as the upcoming targets); and (2) 50% of invalid cues (in the opposite location as the upcoming targets). The cue-target onset asynchronies (CTOA) varied to 0, 400, or 800 ms, and the duration between the offset of the target circles and onset of the next trial (inter trial interval, ITI) varied randomly between 2,000 and 12,000 ms. The task consisted of 4 blocks of 48 test trials each. A total of 192 trials were composed with 96 trials for invalid-cue condition and 96 for valid-cue condition. During the task, eye-movements for all participants were recorded with an eye-tracking device as an overt attention measurement. The first block was the baseline phase with no feedback on both eye-feedback and the control conditions. Different formation of the blocks was used in the intervention phase (1st post-intervention) with feedback in the eye-feedback condition and without feedback in the control condition.

Prior to the game task, users were instructed that the fixation and movement of their gaze served as the click and move of the mouse, a traditional input device. The users were also asked to move their gaze when a target or cue appeared on the screen and make sure to return their gaze back to the car. The users received visual and auditory feedback throughout the game depending on the rate of gaze response recorded by the eye-tracking device. In other words, users were provided constant real-time feedback on the result whether each trial was faster or slower compared to baseline. When the gaze response was faster than the baseline, the screen flashed with the green color (visual feedback), a warning beep was played (auditory feedback), and the car speed increased. However, when the response was slower, a red flash appeared, a different warning beep was provided, and the car speed decreased. At the end of each block, the screen showed the response rate of participants' gaze movement compared to the baseline. In sum, participants in the feedback condition received feedback throughout the game, but participants in the control condition were not provided with two main feedback of the game: (1) real-time verification of the user's gaze position; (2) the response rate to gaze movement.

### Apparatus

The ANT-R was presented on a desktop computer using E-Prime™ 2.0 (Psychology Software Tools, Pittsburgh, PA) on a 23-inch wide monitor. RTs were collected and stored by the desktop computer using a keyboard.

Eye-movements for all participants were recorded with an eye-tracking device (Tobii TX300, Tobii Technology AB, Danderyd, Sweden) at the sampling rate of 300 Hz and the maximum total system latency of 10 ms. It integrates the infrared sensors and the camera. Each participant was seated 70 cm in front of a 23-inch wide monitor (1,920 × 1,080), and the eye tracker allowed the participants to naturally move their heads and eyes without any attached sensors. Eye-movements that were stable for at least 80 ms within the visual angle of 1.4° were defined as fixations (46). The eye-tracking equipment was calibrated for all participants by presenting five dots on the screen, and then the attention training task started. The software (Tobii studio, Tobii Technology AB, Danderyd, Sweden) provided a variety of gaze information, such as the participants' focus and latency to fixation, duration of fixated attention, and so on.

### Procedure

As the participants arrived at the laboratory, they were given a brief instruction regarding the experiment and their rights as research participant; then they filled in the consent form that was approved by the institutional review board of Chung-Ang University (No. 1041078-202001-HR-009-01). The participants were then randomly assigned to either the eye-feedback or the control condition. The study involved three intervention visits. The first intervention visit was conducted to determine study eligibility and to collect baseline data before initiating the intervention period. Therefore, the participants were asked to administer the ANT-R as a covert attention measurement of the baseline phase. After a short break, the participants were instructed to engage with the attention training for ~25 min. Prior to attention training, a total of 24 trials were administered as practice trials to ensure that the participants properly understood how to use the intervention. Then participants performed attention training according to each condition. After the task, the participants were asked to administer the ANT-R as a covert attention measurement after the 1st post-intervention. Also, they were asked to complete a clinical interview and to fill in questionnaires; finally, they also received instructions for the next intervention visits.

At the second intervention visit, the participants did attention training without the ANT-R in both conditions and were given instructions for the next intervention visit (2nd post-intervention). At the last and the third visit, the participants were asked to do attention training in each condition (3rd post-intervention). Afterwards, they performed the ANT-R as a covert attention measurement of last post-intervention. All three visits were organized within the minimum period of 3 days to the maximum period of seven days. Afterwards, participants were debriefed about the experiment and received 50,000 won (ca. 50 USD) as a reward. In addition, all participants were asked not to share any information with anyone who might participate in the experiment after them.

### Data Analysis

For data analysis, a chi-square test and an independent *t*-test to analyze the differences in the characteristics between the eye-feedback and the control groups were performed. Prior to analyzing the ANT-R, mean RT and error rate for each condition were calculated. The attentional network index in RT and the error rate were computed using the definitions described above for Table 1. In the eye-feedback task, mean latency to fixation for each condition was collected using the eye-tracking system. Prior to the analysis of the task, error trials were excluded from the calculations of latency to fixation for attentional effects. The ability to attention engagement representing the selectively attend to specific information was measured by the latency to fixate the target from fixation in valid conditions. The ability to attention disengagement representing disengagement of focus from current target and shifts of focus to another target was measured by the latency to fixate target from fixation in invalid conditions.

**Table 1:** Demographic and clinical characteristic for eye-feedback and control group.

	**Group**		
**Measure**	**Eye-feedback (*N* = 19)**	**Control (*N* = 19)**	**Test statistics (t/χ2)**
Age (years)	21.21 (2.30)	20.68 (1.70)	0.80
Sex (male/female)	7/12	8/11	0.74
**BAARS-IV**			
ADHD IN	17.53 (2.10)	18.24 (2.39)	0.95
ADHD H-I	15.00 (2.73)	15.41 (3.06)	0.43
SCT	25.32 (2.73)	24.12 (1.65)	1.57
ACI	17.95 (3.96)	16.78 (3.89)	0.91
BDI-II	12.68 (8.51)	11.89 (9.10)	0.27
BAI	30.74 (8.51)	25.42 (15.19)	1.07

*Mean (standard deviation); SCT, Sluggish Cognitive Tempo; ADHD, Attention-Deficit/Hyperactivity Disorder; Age, years, BAARS-IV, Barkley Adult Attention-deficit/Hyperactivity Disorder Rating Scale IV; IN, Inattentiveness; H-I, Hyperactivity and impulsivity; ACI, Adult Concentration Inventory; BDI-II, Beck Depression Inventory-II; BAI, Beck Anxiety Inventory*.

In order to investigate the effects of the eye-feedback in individuals with SCT, we used a two-factor mixed design with group as the between-subject factor, and phase as the within-subjects factor. In order to investigate eye-movement data as an overt attention measurement, we conducted 2 (group: eye-feedback, control) × 4 (phase: baseline, 1st post-intervention, 2nd post-intervention, 3rd post-intervention) analysis. In order to examine the ANT-R as a covert attention measurement, we conducted 2 (group: eye-feedback, control) × 3 (phase: baseline, 1st post-intervention, 3rd post-intervention) analysis. Additionally, whenever there was a significant interaction effect, *post-hoc* tests were performed to examine interactions in more detail, and degrees of freedom were adjusted with the Greenhouse-Geisser epsilon to correct for violations of the sphericity assumption. All statistical data were analyzed using SPSS 17.0 for Windows.

## Results

### Demographic and Clinical Characteristics

Table 1 shows the characteristics of the participants analyzed in the present study. There were non-significant differences in mean age [*t*_(36)_ = 0.80, *n.s*.], proportion of sex [$\chi_{\left(1\right)}^{2}$ = 0.74, *n.s*.], ADHD inattention [*t*_(36)_ = 0.95, *n.s*.], ADHD hyperactive-impulse [*t*_(36)_ = 0.43, *n.s*.], SCT [*t*_(36)_ = 0.1.57, *n.s*.], ACI [*t*_(36)_ = 0.91, *n.s*.], BDI-II [*t*_(36)_ = 0.27, *n.s*.], and BAI [*t*_(36)_ = 1.07, *n.s*.] between the eye-feedback and the control groups. In addition, in order to investigate whether the BAARS-IV was associated with the ACI, a series of bivariate correlational analyses were conducted. Analyses revealed the ACI was positively correlated with SCT symptoms of the BAARS-IV [*r* = 0.61, *p* < 0.05]. There was non-significant correlation between the ACI and ADHD inattention of the BAARS-IV [*r* = 0.25, *n.s*.], the ACI and ADHD hyperactive-impulse of the BAARS-IV [*r* = 0.11, *n.s*.]

### Comparison of Eye-Movements Results Between Eye-Feedback and Control Group

Table 2 shows the changes in mean latency to fixation and standard deviation for each phase between the eye-feedback and the control group.

**Table 2:** Results of eye-movements between eye-feedback and control group.

	**Group**		
**Phase**	**Eye-feedback (*N* = 19)**	**Control (*N* = 19)**	**Test statistics (F)**
**ENGAGE ATTENTION**			
Baseline	223.79 (107.06)	242.84 (156.21)	3.34^*^
1st post-intervention	216.65 (142.67)	271.07 (166.48)	
2nd post-intervention	196.28 (127.66)	359.82 (263.29)	
3rd post-intervention	173.81 (119.81)	311.74 (252.69)	
**DISENGAGE ATTENTION**			
Baseline	584.89 (108.45)	566.42 (63.78)	3.24^*^
1st post-intervention	539.11 (88.42)	533.75 (91.32)	
2nd post-intervention	523.16 (86.28)	595.53 (164.12)	
3rd post-intervention	504.04 (50.17)	585.05 (142.65)	

* *p < 0.05; Mean (standard deviation) in milliseconds (ms); SCT, Sluggish Cognitive Tempo; ADHD, Attention-Deficit/Hyperactivity Disorder*.

### Comparison of the Attention Engagement Results Between Eye-Feedback and Control Group

Results of analyses in order to examine within group differences showed that there was a significant effect regarding the phase in the eye-feedback group [*F*_(3, 54)_ = 3.06, *p* < 0.05, η^2^ = 0.15], while non-significant effect was shown in the control group [*F*_(3, 54)_ = 2.11, *n.s*.]. In a *post-hoc* test among phases, there were significant phase differences between the 3rd post-intervention and other two phases (baseline, 1st post-intervention). Results indicated that an increase in efficiency of attention engagement was shown only in the eye-feedback group, and this effect may become more prominent as the attention training repeats.

**Figure 4:**
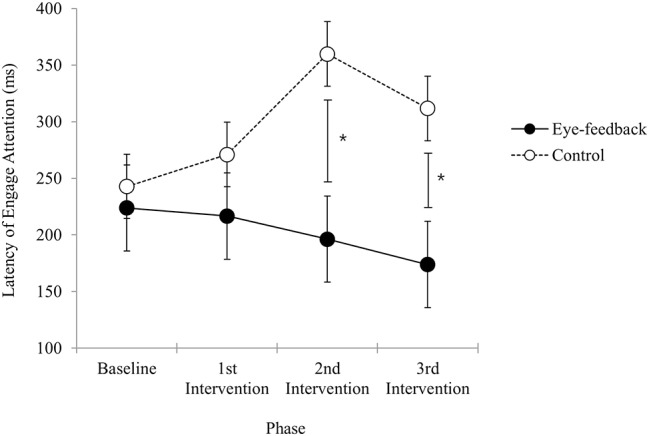
Latency of engage attention. The figure shows change in the attention engagement results across sessions between the eye-feedback and the control group. The asterisk (*) indicates statistically significant difference at *p* < 0.05. Error bars indicate standard error of the mean.

There was a significant interaction on the group and the phase [*F*_(2.45, 88.19)_ = 3.34, *p* < 0.05, η^2^ = 0.09]. With a *post-hoc* test at each phase, non-significant group difference was observed at the baseline phase [*t*_(36)_ = 0.44, *n.s*.] and the 1st post-intervention phase [*t*_(36)_ = 1.08, *n.s*.]. However, there were significant group differences at the 2nd post-intervention phase [*t*_(36)_ = 2.44, *p* < 0.05] and 3rd post-intervention phase [*t*_(36)_ = 2.15, *p* < 0.05]. Results indicated that the attention engagement at the baseline did not differ between the eye-feedback and the control groups, and a similar result was obtained until the 1st post intervention phase. While at the 2nd post-intervention and the 3rd post-intervention phase, the attention engagement of the eye-feedback group was faster than the control group (see Figure 4). There was non-significant effect on the group [*F*_(1, 36)_ = 3.96, *n.s*.], indicating that statistically insignificant differences between the two groups were found. There was non-significant main effect on the phase [*F*_(2.45, 88.19)_ = 1.11, *n.s*.], indicating that statistically insignificant differences between the four phases were found.

### Comparison of the Attention Disengagement Results Between Eye-Feedback and Control Group

Results of analyses in order to examine within group differences showed that there was a significant effect regarding the phase in the eye-feedback group [*F*_(3, 54)_ = 4.82, *p* < 0.01, η^2^ = 0.21], while non-significant effect was found in the control group [*F*_(3, 54)_ = 2.11*, n.s*.]. With a *post-hoc* test among phases, there were significant phase differences between the baseline and the other three phases (1st post-intervention, 2nd post-intervention, 3rd post-intervention). Results indicated that an increase in efficiency of attention disengagement was shown only in the eye-feedback group, and this effect was consistent after the 1st post-intervention.

**Figure 5:**
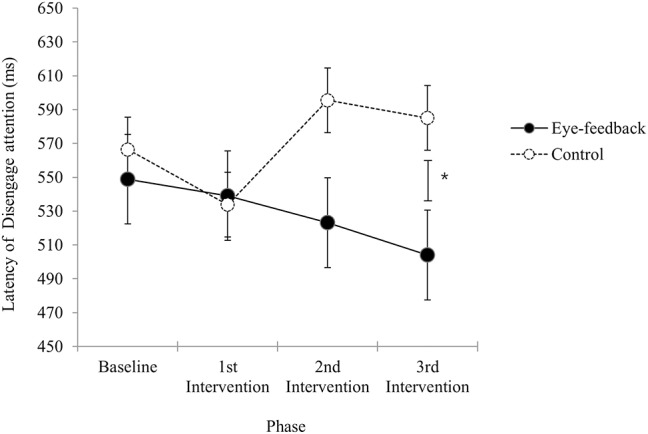
Latency of disengage attention. The figure shows change in the attention disengagement results across sessions between the eye-feedback and the control group. The asterisk (*) indicates statistically significant difference at *p* < 0.05. Error bars indicate standard error of the mean.

There was a significant interaction between the group and the phase [*F*_(2.45, 87.06)_ = 3.24, *p* < 0.05, η^2^ = 0.08]. With a *post-hoc* test at each phase, non-significant group differences were observed at the baseline phase [*t*_(36)_ = 0.64, *n.s*.], the 1st post-intervention phase [*t*_(36)_ = 0.18, *n.s*.], and the 2nd post-intervention phase [*t*_(36)_ = 1.70, *n.s*.]. However, there was a significant group differences at the 3rd post-intervention phase [*t*_(36)_ = 2.34, *p* < 0.05]. Results indicated that the attention disengagement at the baseline did not differ between the eye-feedback and the control groups, and a similar result was obtained until the 2nd post-intervention phase. While at the 3rd post-intervention phase, the attention disengagement of the eye-feedback group was faster than the control group (see Figure 5). There was non-significant main effect on the group [*F*_(1, 36)_ = 1.88*, n.s*.], indicating that statistically non-significant differences between the two groups were found. There was non-significant main effect on the phase [*F*_(2.45, 87.06)_ = 1.46, *n.s*.], indicating that statistically non-significant differences between the four phases were found.

### Comparison of ANT-R Results Between Eye-Feedback and Control Group

Table 3 shows changes in attentional network index in RT for each phase between the eye-feedback and the control group.

**Table 3:** Results of ANT-R between eye-feedback and control group.

	**Group**		
**Phase**	**Eye-feedback (*N* = 19)**	**Control (*N* = 19)**	**Test Statistics (F)**
**ALERTING**			
Baseline	58.74 (36.05)	*65.37 (35.14)*	0.50^*^
1st post-intervention	72.89 (31.56)	68.10 (33.55)	
3rd post-intervention	58.47 (34.65)	70.58 (40.89)	
**VALIDITY**			
Baseline	26.00 (45.02)	21.37 (26.64)	1.20^*^
1st post-intervention	16.84 (40.09)	9.37 (26.40)	
3rd post-intervention	−0.42 (18.46)	13.26 (30.86)	
**ENGAGING**			
Baseline	−24.16 (29.71)	−19.05 (21.47)	0.52^*^
1st post-intervention	−23.84 (27.48)	−22.05 (30.89)	
3rd post-intervention	−21.37 (19.13)	−27.74 (21.89)	
**DISENGAGING**			
Baseline	50.16 (39.41)	40.42 (28.94)	3.63^*^
1st post-intervention	41.11 (34.61)	31.00 (37.01)	
3rd post-intervention	20.95 (25.71)	44.16 (16.23)	
**EXECUTIVE CONTROL**			
Baseline	136.95 (53.65)	122.68 (32.57)	0.85^*^
1st post-intervention	125.89 (57.24)	119.26 (38.79)	
3rd post-intervention	107.37 (29.89)	113.26 (31.14)	

* *p < 0.05; Mean (standard deviation) in milliseconds (ms); SCT, Sluggish Cognitive Tempo; ADHD, Attention-Deficit/Hyperactivity Disorder*.

### Comparison of the Alerting Network Index Results Between Eye-Feedback and Control Group

There was non-significant interaction between the group and the phase [*F*_(2, 72)_ = 0.65, *n.s*.], indicating that the two groups did not differ in the benefit of the target response speed because of alerting network at each phase. There was non-significant main effect on the group [*F*_(1, 36)_ = 0.66, *n.s*.], indicating that statistically non-significant differences between the two groups were found. There was non-significant main effect on the phase [*F*_(2, 72)_ = 1.03, *n.s*.], indicating that statistically non-significant differences between the three phases were found.

### Comparison of the Engaging Index Results Between Eye-Feedback and Control Group

There was non-significant interaction between the group and the phase [*F*_(2, 72)_ = 0.57, *n.s*.], indicating that the two groups did not differ in the benefit of target response because of engaging attention at each phase. There was non-significant main effect on the group [*F*_(1, 36)_ = 0.01, *n.s*.], indicating that statistically non-significant differences between the two groups were found. There was non-significant main effect on the phase [*F*_(2, 72)_ = 0.14, *n.s*.], indicating that statistically non-significant differences between the three phases were found.

### Comparison of the Disengaging Index Results Between Eye-Feedback and Control Group

Results of analyses in order to examine within group differences showed that there was a significant effect regarding the phase in the eye-feedback group phase [*F*_(2, 36)_ = 4.01, *p* < 0.05, η^2^ = 0.18], while there was non-significant effect in the control group [*F*_(2, 36)_ = 1.02, *n.s*.]. With a *post-hoc* test among phases, there were significant phase differences between the 3rd post-intervention and other two phases (baseline, 1st post-intervention). Results indicated that an increase in efficiency of orienting network related to disengaging attention was only shown in the eye-feedback group, and this effect may improve as the attention training repeats.

There was a significant interaction between the group and the phase [*F*_(2, 72)_ = 3.63, *p* < 0.05, η^2^ = 0.09]. With a *post-hoc* test at each phase, non-significant group difference was observed at the baseline phase [*t*_(36)_ = 0.87, *n.s*.] and the 1st post-intervention phase [*t*_(36)_ = 0.87, *n.s*.]. However, there were significant group differences at the 3rd post-intervention phase [*t*_(36)_ = 3.33, *p* < 0.05]. Results indicated that the disengaging index at the baseline did not differ between the eye-feedback and the control groups, and a similar result was obtained up until the 1st post intervention phase. While at the 3rd post-intervention phase, the disengaging index of the eye-feedback group was lower than the control group. There was a non-significant main effect on the group [*F*_(1, 36)_ = 0.04, *n.s*.], indicating that statistically non-significant differences between the two groups were found. There was non-significant main effect on the phase [*F*_(2, 72)_ = 1.72, *n.s*.], indicating that statistically non-significant differences between the three phases were found.

### Comparison of the Executive Control Network Index Results Between Eye-Feedback and Control Group

There was a significant main effect on the phase [*F*_(2, 72)_ = 3.21, *p*< *0.05*, η^2^ = *0.08*]. With a *post-hoc* test among phases, there were significant phase differences between the 3rd post-intervention and the baseline phase. Results indicated that increases in efficiency of executive control network become better as the attention training repeats regardless of groups. There was non-significant interaction between the group and the phase [*F*_(2, 72)_ = 0.86, *n.s*.], indicating that the two groups did not differ in the cost of target response because of the flanker conflict effect at each phase. There was non-significant main effect on the group [*F*_(1, 36)_ = 0.24, *n.s*.], indicating that statistically non-significant differences between the two groups were found.

## Discussion

The present study developed and assessed a preliminary attention training program targeting the orienting network based on a real-time eye-gaze feedback using an eye-tracking system in order to improve dysfunction of attentional networks in individuals with SCT. As a result, those individuals with SCT who were assigned to the eye-feedback condition showed more improvement in engaging and disengaging their attention using the measurement of eye-movements than those in the control condition. In addition, the eye-feedback group showed more improvement than the control group only in the efficiency of orienting related to disengaging attention using the measurement of the ANT-R. Additionally, in both groups, there was an increase in efficiency of the executive control network after the repeated attention training.

The major finding of the present study is that attention training based on eye-feedback could enhance both engagement and disengagement of overt attention in individuals with SCT. In line with previous research (7, 8), this supports our hypothesis that efficiency of attention could be improved through repeated practice. There are three possible explanations of these results. First, the eye-feedback was developed to provide repeated attention training using Pro-SEM based on behavior plasticity. The task consisted of 4 blocks, each with 48 trials, for a total of 192 trials (~25 min) and was designed to be repeated three times. The results of the present study indicated that increases in efficiency of attention engagement and disengagement may become better as attention training repeats. These results are in line with previous findings that repeated training of the SEM can produce changes in oculomotor performance, leading to an improvement in overt attention (13, 17, 20). Second, the eye-feedback was developed to improve specifically the orienting network. In order to directly and overtly measure attention engagement and disengagement using an eye-tracking system, a modified Posner spatial cueing paradigm previously used in a variety of attention orienting studies was used. Therefore, the eye-feedback could be used to not only assess the overt orienting on engagement and disengagement of attention, but also to improve these networks through repeated training. Finally, the eye-feedback was developed to provide real-time constant eye-gaze feedback during repeated training, and this was a distinctive characteristic of the task. In the present study, an increase in efficiency of engagement and disengagement on overt attention was observed in the eye-feedback group, but not in the control group. These results suggest that feedback could improve performance by reducing uncertainty and providing information to focus on correct, incorrect, or both (28). Also, these results are consistent with previous studies that suggested that an adaptive type of attention training (which can provide feedback during a task responsive performance) is more effective than a non-adaptive type of attention training. Therefore, in our study, a greater improvement among individuals was observed when feedback was given (7, 27). Eye-feedback is such a modified version of bio-feedback, similar to the neuro-feedback and fMRI-feedback, which is meaningful in that the task focused directly on improving visual attention.

Another meaningful result of the present study is that attention training based on eye-feedback could enhances only disengagements on covert attention in individuals with SCT. However, since an increase in efficiency of engagements on covert attention was not observed, hypotheses of the present study are partially supported. Therefore, the controversy about the relation between covert and overt attention remains. Several previous studies suggested that covert and overt shift of attention are independent of one another, which is known as the modular theory of attention (23, 47). By contrast, other studies results suggested that covert and overt attention cannot be independently changed, which is known as the premotor theory of attention (48). In the present study, since eye-feedback was overt attention training based on eye-feedback, it could improve efficiency of both engagement and disengagement on overt attention. There was also an increase in efficiency of disengagement on covert attention, but not in efficiency of engagement on covert attention. Although further studies are needed, there appears to be a link between covert shift of attention and eye-gaze process during disengagement, and these results support that training one might induce benefits of the other (26). Also, these results support the intermediate view of those theories that covert and overt attention may elicit both common and different regions of brain activation (49).

Interestingly, although attention training based on the eye-feedback did target the executive control network, increases in efficacy of the network become better as the attention training repeats regardless of groups. There are several possible explanations for this result. First, it is possible that the practice effect occurred in the executive control network only. This idea is consistent with previous reports of practice effects on the alerting and executive control networks in the ANT, as the difference of the scores changed significantly between sessions (32, 50). Second, it is possible that conducting repeated attention training might induce improvement in the executive control network. That is, although the cognitive load was low, the task requirement of focusing attention on any task for a substantial amount of time appeared to improve executive control (51), which is also supported by a previous finding that repetitive saccade execution can improve attention control (52).

The present study has several limitations. First, although we observed that eye-feedback can enhance attentional functions through three repeated practices, those three practices may not have been sufficient to confirm the practice effect. Our results suggest that, in both conditions, an increase in efficiency of the orienting network became larger after repetitive training, even if efficiency of the executive function improved. Second, we cannot investigate whether the attention training could transfer to SCT or ADHD symptoms because we did not measure SCT or ADHD symptoms after training. Thus, further research should examine the attention training based on eye-feedback in order to elucidate the improvement in not only the orienting network, but also other issues, such as SCT or ADHD symptoms, academic functioning, internalizing symptoms, and quality of life. Therefore, the study needs longitudinal follow-up and requires to examine other outcomes and functional impairment. Finally, the study needs to be replicated in better characterization of the clinical groups included—in relation to for instance comorbidities and intellectual abilities.

In summary, the results of the present study preliminary indicate that repeated attention training using the eye-feedback could improve orienting on both covert and overt attention in individuals with SCT. Our findings also provide a novel intervention targeting attentional difficulties among individuals with SCT. Specifically, the use of the eye-feedback as attention training in a promising means of improving efficiency of the orienting network in clinical settings.

## Data Availability Statement

The datasets generated for this study are available on request to the corresponding author.

## Ethics Statement

The experiment was approved by the institutional review board in Chung-Ang University. All of the participants signed an informed consent that had been approved by the institutional review board in Chung-Ang University. The patients/participants provided their written informed consent to participate in this study.

## Author's Note

This paper is a condensed version of KK's doctoral thesis.

## Author Contributions

KK, YL, and J-HL: conceived the experiment. KK: designed the experimental task. KK and YL: participants' data acquisition. KK and YL: data analysis. KK and J-HL: data interpretation. KK, YL, and J-HL: drafting of the manuscript. All the authors revised the manuscript critically and gave the final approval of the version to be published.

### Conflict of Interest

The authors declare that the research was conducted in the absence of any commercial or financial relationships that could be construed as a potential conflict of interest.
